# Risk-associated management disparities in acute myocardial infarction

**DOI:** 10.1038/s41598-021-03742-6

**Published:** 2021-12-29

**Authors:** Kai M. Eggers, T. Jernberg, B. Lindahl

**Affiliations:** 1 Department of Medical Sciences, Cardiology, Uppsala Clinical Research Center, Uppsala University, 751 85 Uppsala, Sweden; 2 Department of Clinical Sciences, Cardiology, Karolinska Institute, Danderyd Hospital, Stockholm, Sweden

**Keywords:** Myocardial infarction, Cardiology

## Abstract

Despite improvements in the treatment of myocardial infarction (MI), risk-associated management disparities may exist. We investigated this issue including temporal trends in a large MI cohort (n = 179,291) registered 2005–2017 in SWEDEHEART. Multivariable models were used to study the associations between risk categories according to the GRACE 2.0 score and coronary procedures (timely reperfusion, invasive assessment ≤ 3 days, in-hospital coronary revascularization), pharmacological treatments (P2Y12-blockers, betablockers, renin–angiotensin–aldosterone-system [RAAS]-inhibitors, statins), structured follow-up and secondary prevention (smoking cessation, physical exercise training). High-risk patients (n = 76,295 [42.6%]) experienced less frequent medical interventions compared to low/intermediate-risk patients apart from betablocker treatment. Overall, intervention rates increased over time with more pronounced increases seen in high-risk patients compared to lower-risk patients for in-hospital coronary revascularization (+ 23.6% vs. + 12.5% in patients < 80 years) and medication with P2Y12-blockers (+ 22.2% vs. + 7.8%). However, less pronounced temporal increases were noted in high-risk patients for medication with RAAS-blockers (+ 8.5% vs. + 13.0%) and structured follow-up (+ 31.6% vs. + 36.3%); *p*_interaction_ < 0.001 for all. In conclusion, management of high-risk patients with MI is improving. However, the lower rates of follow-up and of RAAS-inhibitor prescription are a concern. Our data emphasize the need of continuous quality improvement initiatives.

## Introduction

The past decades have seen considerable improvements in the management of patients with myocardial infarction (MI). This applies to the more frequent use of invasive treatments but also of medications with proven prognostic benefit^1–3^. Guidelines stress the importance of formal risk assessment to identify MI patients at highest risk for poor outcome in order to customize management. However, several studies demonstrate an underutilization of beneficial interventions in high-risk patients^1,4–6^, a phenomenon labelled treatment-paradox. The clinical dimension of this problem is amplified by the fact that high-risk patients often derive greater treatment benefit compared to lower-risk patients. While efforts have been made to reduce these disparities^1^, they still appear to exist. Several studies investigating this important issue however, only focused on invasive treatments^6,7^, and there is a paucity of data on temporal changes in management patterns in contemporary real-world cohorts^1^.

The GRACE 2.0 score is a well-validated tool for the prediction of all-cause mortality in acute coronary syndrome^7^. The score employs non-linear functions to model risk based on age, heart rate, systolic blood pressure, creatinine as continuous variables, and Killip class, cardiac arrest at admission, ST-segment deviation and elevation of circulating biomarkers of myocardial necrosis as categorical variables. The GRACE 2.0 score has recently been given a class IIa recommendation in European guidelines^8^.

Using the GRACE 2.0 score, the aims of the present study were (1) to investigate risk-associated management disparities in a large cohort of MI patients during the course of disease, from early invasive assessment to secondary prevention, and (2) to assess whether the magnitude of potentially existing treatment disparities might have changed over time. We hypothesized that management of MI patients still differs among patient categories at different levels of risk whilst the broad uptake of guideline-based management recommendations^1–3^ has reduced the dimension of this issue.

## Material and methods

### Study population

This study is part of the TOTAL-AMI (Tailoring of Treatment in All comers with Acute Myocardial Infarction) project. The primary aim of TOTAL-AMI is to study the mechanisms and implications of different MI subtypes^9^ and of comorbidities (e.g. chronic obstructive pulmonary disease, atrial fibrillation, renal dysfunction) in MI. TOTAL-AMI uses data from SWEDEHEART (Swedish Web-system for Enhancement and Development of Evidence-based care in Heart disease Evaluated According to Recommended Therapies) which is a registry collecting data from patients admitted to Swedish coronary care units or other specialized facilities because of suspected acute coronary syndrome. In subregistries, data on in-hospital management (Register for Information and Knowledge about Swedish Heart Intensive Care Admissions [RIKS-HIA]), invasive procedures (Swedish Coronary Angiography and Angioplasty Registry [SCAAR]) and follow-up 6–10 weeks after hospital discharge (Secondary Prevention after Heart Intensive care Admission Registry [SEPHIA]) is aggregated. SWEDEHEART provides almost nationwide coverage and lifelong follow-up. Upon hospital admission, patients receive information about SWEDEHEART, have the right to deny participation and to get their data erased upon request. Written informed consent is not required according to Swedish law.

The population for the present study included all MI patients admitted between January 2005 and May 2017 with complete data necessary for the calculation of the GRACE 2.0 score 7. Only the first registered MI during the study period was considered. The follow-up cohort consisted of MI patients aged < 75 years, managed at hospitals participating in SEPHIA. Patients at higher ages had no scheduled follow-up within the SEPHIA framework during the study period. For surviving patients not participating in the 6–10 week follow-up, a fictive follow-up date at 60 days from hospital admission was created in the dataset. This corresponds to the mean timepoint from admission at which follow-up took place in those who participated (60 ± 14 days). Non-participants who had died before this date were censored.

All data had been made pseudonymized before the statistical analyses. The study was conducted according to the principles of the 1975 Declaration of Helsinki and had been approved by the Regional Ethical Review Board in Stockholm (2012/60-31/2).

### Investigated medical interventions

We studied the rates of the following medical interventions that are partly incorporated as quality/performance measures in the SWEDEHEART quality index^10^:

- *Coronary procedures:* early reperfusion (thrombolysis ≤ 30 min or percutaneous coronary intervention [PCI] ≤ 90 min from first ECG) in ST-elevation MI (STEMI), coronary angiography ≤ 3 calendar days from admission in non-ST-elevation MI (NSTEMI), in-hospital PCI or coronary artery bypass grafting (CABG);
- *Pharmacological treatments:* discharge medication with P2Y12-blockers, betablockers, renin–angiotensin–aldosterone-system (RAAS)-inhibitors or statins;
- *Follow-up and secondary preventive measures:* Participation in the 6–10 week follow-up, self-reported smoking cessation, participation in exercise training within a cardiac rehabilitation programme.

Since some treatment decisions might have been affected by the presence or absence of specific comorbidities or contraindications, we applied the following intervention-specific exclusion criteria:

- *Total cohort:* dementia;
- *Coronary angiography* ≤ 3 days *(NSTEMI):* hemoglobin < 80 g/L, estimated glomerular filtration rate (eGFR; CKD-EPI equation) < 20 mL/min/1.73 m^2^;
- *In-hospital PCI/CABG:* hemoglobin < 80 g/L, eGFR < 20 mL/min/1.73 m^2^;
- *Discharge medication with P2Y12-blockers:* hemoglobin < 80 g/L;
- *Discharge medication with betablockers:* heart rate < 50/min;
- *Discharge medication with RAAS-inhibitors:* eGFR < 20 mL/min/1.73 m^2^, left-ventricular ejection fraction > 0.50 in patients without concomitant diabetes, hypertension or known heart failure.

### Statistical analysis

All continuous variables were skewed and are reported as medians with 25^th^ and 75^th^ percentiles. Categorical variables are expressed as frequencies and percentages. The prognostic accuracy of the GRACE 2.0 score was estimated by the calculation of the c-statistics. Kaplan–Meier curves were plotted to illustrate the occurrence of death across risk cohorts defined by estimated probabilities of 1-year all-cause mortality of < 3% (low risk), 3–8% (intermediate risk) and > 8% (high risk) according to the GRACE 2.0 score.

Multivariable logistic regressions were used to investigate the associations between risk categories and the use of medical interventions. Adjustment was made for admission year, hospital, sex, current smoking, diabetes, previous MI, previous coronary revascularization, previous heart failure, previous stroke, atrial fibrillation upon admission, chronic obstructive pulmonary disease, previous or present cancer, and peripheral artery disease. Since the decision to perform PCI/CABG depends on the extent of coronary stenoses, information on coronary findings (categorized as normal or non-occlusive disease, 1–2 vessel obstructive disease, 3-vessel obstructive disease/left main stem and inconclusive findings) was included in models investigating in-hospital PCI/CABG. Similarly, models investigating discharge medication with P2Y12-blockers were additionally adjusted for in-hospital PCI/CABG. Clinical data employed as GRACE 2.0 score components were not considered in the models. Results are presented as odds ratios (OR) with 95% confidence intervals (CI). Since OR may overestimate true effect sizes in case of high intervention rates, we pragmatically focused on those with rates below benchmarks adopted from the SWEDEHEART quality index: ≥ 75% for coronary interventions, ≥ 85% for pharmacological treatments, ≥ 75% for participation in the 6–10 week follow-up, ≥ 60% for smoking cessation and ≥ 50% for participation in exercise training^10^.

Temporal changes in the use of medical interventions were assessed (1) by inclusion of an interaction term between calendar year and risk group in the fully adjusted logistic regression, and (2) by additional adjustment for the two first vs. the two last years of the observation period as explanatory covariates.

In explorative analyses, we repeated all calculations in patients aged < 80 years since treatment decisions tend to be highly individualized in patients at higher age, an entity that also contributes to higher GRACE 2.0 score.

No imputation was performed in case of missing data. In all tests, a two-sided *p* value < 0.05 was considered significant. The software package SPSS 27.0 (SPSS Inc., Chicago, IL) was used for the analyses.

## Results

**Figure 1:**
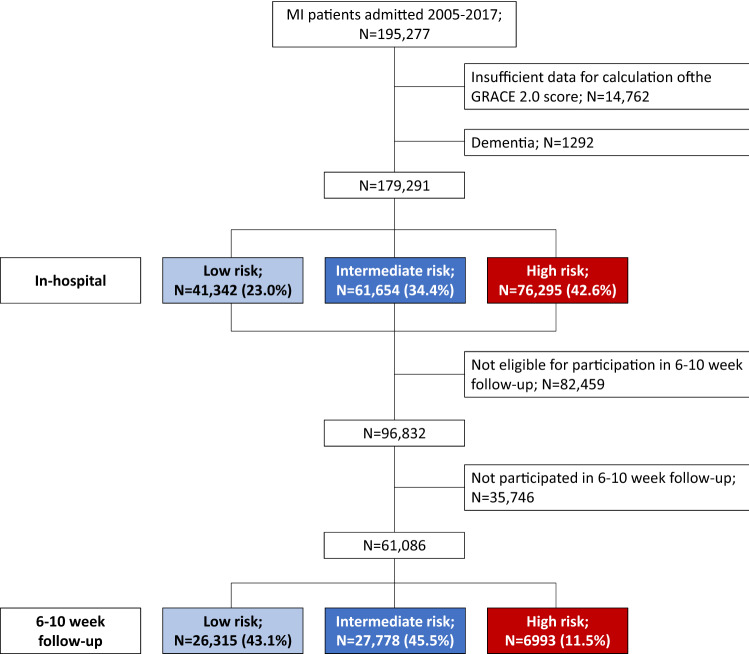
Study flowchart.

The study cohort consisted of 195,277 unique patients with MI. Following exclusions, 41,342 (23.1%), 61.654 (34.4%) and 76,295 (42.6%) patients had low, intermediate and high estimated probabilities of 1-year mortality, respectively. The proportions of low-risk patients and intermediate-risk patients increased by + 2.3% and + 4.0% during the observation period, respectively, whereas the proportion of high-risk patients decreased by -6.1%. Totally 61,086 patients had a follow-up visit at 6–10 weeks. Of these, 26,315 (43.1%), 27,778 (45.5%) and 6993 (11.5%) patients had low, intermediate and high estimated probabilities of 1-year mortality, respectively. An overview of excluded patients and the numbers of patients per GRACE 2.0 score risk category overall and among those who participated in the 6–10 week follow-up is presented in Fig. 1. Further information on clinical characteristics, medical interventions and eligible patients for the respective analyses is presented in Table 1 and Supplementary Table S1. Data on the timing of reperfusion was missing in 15,107 (23.3%) STEMI patients and data on the timing of invasive assessment was missing in 28,531 (28.8%) NSTEMI patients. Excluding patients who did not receive any of these interventions left 8168 (12.6%) STEMI patients with missing data on early reperfusion and 704 (0.7%) NSTEMI patients with missing data on coronary angiography ≤ 3 days. Patients with missing data were older, more likely to be female, had higher prevalence of cardiovascular comorbidities and considerably worse outcome compared to those being included in the main analyses (Supplementary Table S2A and B).

**Table 1:** Clinical characteristics and medical interventions in relation to GRACE 2.0 score categories.

Risk category*	Low risk (n = 41,342)	Intermediate risk (n = 61,654)	High risk (n = 76,295)	Total cohort (n = 179,291)	Missing data	Exclusions
**Risk factors**						
Age (years)	57 (51–63)	69 (64–75)	82 (76–86)	72 (63–81)	–	–
Men	31,439 (76.0%)	41,239 (66.9%)	43,065 (56.4%)	115,743 (64.6%)	–	–
Current smoking	14,407 (34.8%)	13,996 (22.7%)	8406 (11.0%)	36,809 (20.5%)	4	–
Hypertension	15,386 (37.2%)	29,541 (47.9%)	42,697 (56.0%)	87,624 (48.9%)	3	–
Diabetes	5670 (13.7%)	12,294 (19.9%)	19,404 (25.4%)	37,368 (20.8%)	3	–
Hyperlipidemia	8412 (20.4%)	17,283 (28.0%)	22,106 (29.0%)	47,801 (26.7%)	53	–
Body mass index (kg/m^2^)	27.5 (25.0–30.5)	26.6 (24.2–29.4)	25.4 (23.0–28.3)	26.3 (23.9–29.4)	32,648	–
eGFR (mL/min/1.73 m^2^)	93.1 (83.1–100.7)	79.8 (67.1–90.0)	54.5 (39.9–70.8)	74.7 (55.2–89.4	–	–
**Comorbidities**						
Previous MI	4425 (10.7%)	10,240 (16.6%)	20,519 (26.9%)	35,184 (19.6%)	1	–
Previous PCI/CABG	4439 (10.7%)	9195 (14.9%)	12,043 (15.8%)	25,677 (14.3%)	1	–
Heart failure	621 (1.5%)	2574 (4.2%)	10,879 (14.3%)	14,074 (7.8%)	1	–
Atrial fibrillation at admission	568 (1.4%)	3906 (6.3%)	15,142 (19.8%)	19,616 (10.9%)	2	–
Previous stroke	1129 (2.8%)	4301 (7.1%)	10,469 (13.9%)	15,899 (9.0%)	2202	–
Peripheral artery disease	627 (1.5%)	2580 (4.2%)	6929 (9.1%)	10,136 (5.7%)	–	–
COPD	1098 (2.7%)	4214 (6.8%)	7788 (10.2%)	13,100 (7.3%)	–	–
Previous/present cancer	361 (0.9%)	1512 (2.5%)	3522 (4.6%)	5395 (3.0%)	–	–
**Diagnosis**						
NSTEMI	28,694 (69.4%)	37,257 (60.4%)	48,490 (63.6%)	114,441 (63.8%)	–	–
STEMI	12,648 (30.6%)	24,397 (39.6%)	27,805 (36.4%)	64,850 (36.2%)	–	–
**Coronary procedures**						
ICA	36,443 (96.8%)	50,040 (89.9%)	36,321 (56.9%)	122,804 (78.2%)	–	22,204
Early reperfusion (STEMI)	7058 (65.8%)	13,632 (66.4%)	11,199 (60.6%)	31,889 (64.1%)	15,107	–
ICA ≤ 3 days (NSTEMI)	19,367 (77.8%)	20,054 (70.7%)	10,936 (63.0%)	50,357 (71.3%)	28,531	15,299
In-hospital PCI/CABG	28,249 (75.1%)	38,630 (69.4%)	26,834 (42.1%)	93,713 (59.7%)	–	22,204
**Pharmacological treatment**^a^						
P2Y12-blockers	33,564 (89.4%)	46,791 (84.9%)	41,914 (69.3%)	122,269 (79.8%)	179	17,679
Betablockers	35,921 (90.0%)	53,263 (90.2%)	57,447 (86.4%)	146,631 (88.7%)	179	5444
RAAS-inhibitors	16,646 (88.1%)	31,459 (87.5%)	33,212 (78.1%)	81,317 (83.5%)	179	73,541
Statins	39,411 (95.6%)	56,318 (92.5%)	45,959 (72.2%)	145,324 (85.1%)	179	–
**Follow-up and secondary preventive measures**^b^						
Participation in follow-up	26,315 (65.9%)	27,778 (63.6%)	6993 (53.0%)	61,086 (63.1%)	–	–
Smoking cessation^c^	5707 (63.9%)	4718 (62.9%)	1185 (64.3%)	11,610 (63.5%)	23	–
Exercise training	11,226 (42.8%)	11,106 (40.1%)	2571 (37.0%)	24,903 (40.9%)	264	–

*eGFR* estimated glomerular filtration rate, *MI* myocardial infarction, *PCI* percutaneous coronary intervention, *CABG* coronary artery bypass grafting, *COPD* chronic obstructive pulmonary disease, *NSTEMI* non-ST-elevation myocardial infarction, *STEMI* ST-elevation myocardial infarction, *ICA* invasive coronary angiography, *RAAS* renin–angiotensin–aldosterone-system.

*Numbers refer to the total cohort without consideration of patients with missing data or exclusions.

^a^Assessed in in-hospital survivors (n = 171,009).

^b^Assessed in patients eligible for 6–10 week follow-up (n = 96,832).

^c^Assessed in current smokers (upon index hospitalization) participating in the 6–10 week follow-up (n = 18,301).

**Figure 2:**
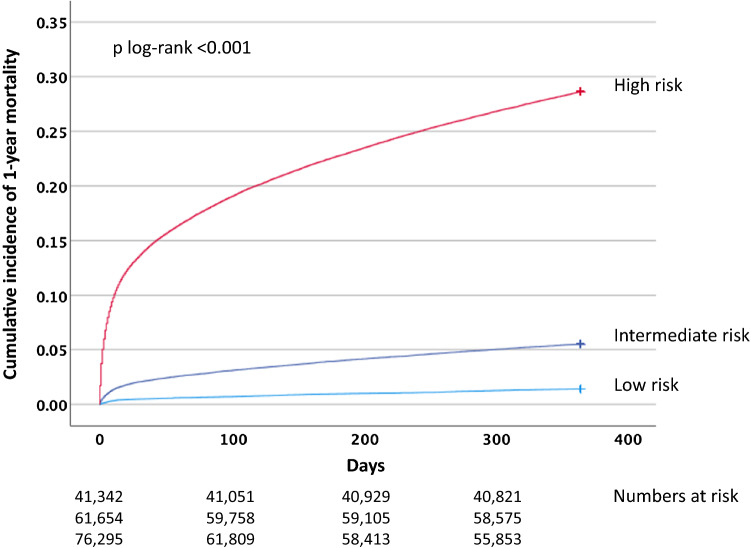
Cumulative incidence of 1-year all-cause mortality across patient cohorts with low, intermediate and high risk.

During 1 year of follow-up, 25,837 (14.4%) patients died (low risk: 585 [1.4%] patients, intermediate risk: 3394 [5.5%] patients, high risk: 21,858 [28.6%] patients). Figure 2 illustrates the cumulative probability of 1-year all-cause mortality across risk cohorts. The overall prognostic accuracy of the GRACE score was high with a c-statistics of 0.829 (95% CI 0.826–0.831).

High risk according to the GRACE 2.0 score was associated with less use of early reperfusion in STEMI, coronary angiography ≤ 3 days in NSTEMI and in-hospital PCI/CABG (Table 2). High-risk patients were less likely to be discharged with P2Y12-blockers and RAAS-inhibitors. Prescription rates of betablockers and statins exceeded 85% in the total population (Table 1) and were thus, not considered in the multivariable models. High-risk patients moreover, less often participated in the 6–10 week follow-up. Among those who attended follow-up, high risk patients less often participated in exercise training and were less likely to have stopped smoking.

**Table 2:** Utilization of medical interventions in high-risk patients.

	n	OR (95% CI)	*p*
**Coronary procedures**			
Early reperfusion (STEMI)	49,225	0.88 (0.85–0.92)	< 0.001
ICA ≤ 3 days (NSTEMI)	70,505	0.69 (0.67–0.72)	< 0.001
In-hospital PCI/CABG	116,883	0.80 (0.76–0.83)	< 0.001
Pharmacological treatments*			
P2Y12-blockers	152,913	0.68 (0.66–0.70)	< 0.001
RAAS-inhibitors	96,820	0.57 (0.55–0.59)	< 0.001
**Follow-up and secondary preventive measures**^a^			
Participation in follow-up	95,564	0.73 (0.70–0.76)	< 0.001
Smoking cessation^b^	18,227	0.79 (0.71–0.88)	< 0.001
Exercise training	60,622	0.87 (0.82–0.91)	< 0.001

Odds ratios refer to comparisons of high-risk patients with low- and intermediate-risk patients, considered as one group.

Analysis adjusted for hospital, admission year, sex, current smoking, diabetes, congestive heart failure, previous myocardial infarction, previous percutaneous coronary intervention/coronary artery bypass grafting, previous stroke, atrial fibrillation upon admission, chronic obstructive pulmonary disease, previous or present cancer, peripheral vascular disease, coronary findings (in-hospital PCI/CABG only) and in-hospital PCI/CABG (P2Y12-blockers only).

*OR* odds ratio, *CI* confidence interval, *STEMI* ST-elevation myocardial infarction, *ICA* invasive coronary angiography, *NSTEMI* non-ST-elevation myocardial infarction, *PCI* percutaneous coronary intervention, *CABG* coronary artery bypass grafting, *RAAS* renin–angiotensin–aldosterone-system.

*Assessed in in-hospital survivors (n = 171,009).

^a^Assessed in patients eligible for 6–10 week follow-up (n = 96,832).

^b^Assessed in current smokers (upon index hospitalization) participating in the 6–10 week follow-up (n = 18,301).

**Figure 3:**
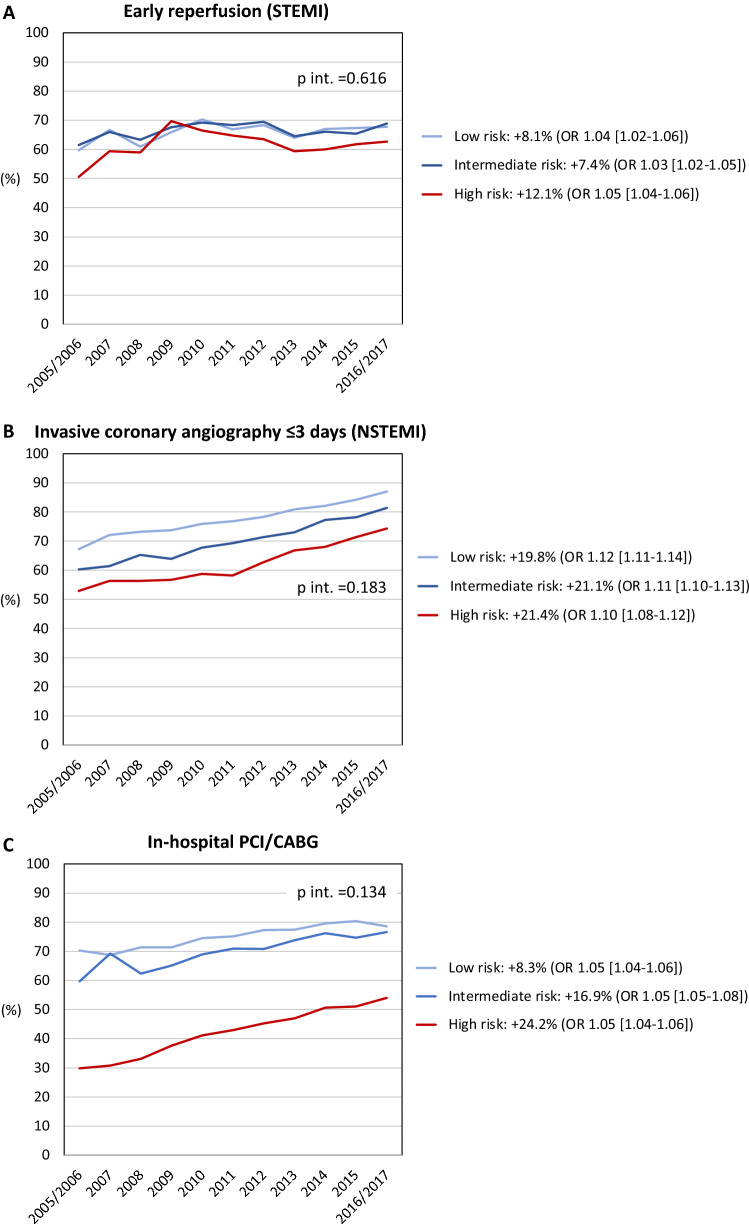
Temporal changes in coronary interventions. (**A**) Early reperfusion in STEMI; (**B**) coronary angiography ≤ 3 days in NSTEMI; (**C**) in-hospital PCI/CABG. Percentages refer to changes in the rates of coronary interventions from 2005/2006 to 2016/2017. *p* int. refers to the interaction between year of admission and risk group on the utilization of coronary interventions. Odds ratios (OR; with 95% confidence intervals) describe the adjusted associations of the year of admission (2005/2006 vs. 2016/2017) with coronary interventions. *STEMI* ST-elevation myocardial infarction, *NSTEMI* non-ST-elevation myocardial infarction, *PCI* percutaneous coronary intervention, *CABG* coronary artery bypass grafting.

**Figure 4:**
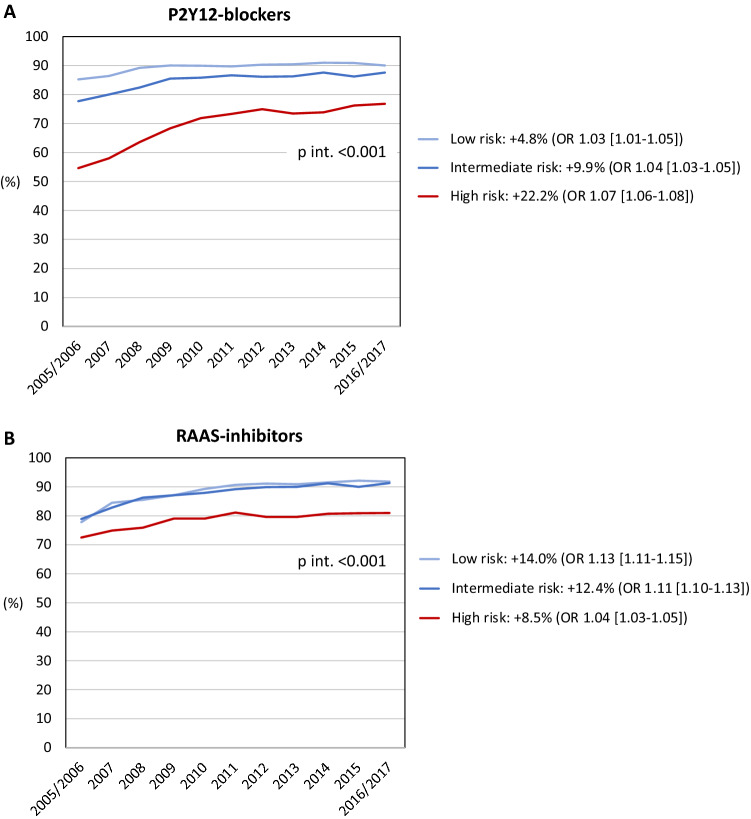
Temporal changes in pharmacological treatments at discharge. (**A**) P2Y12-blockers; (**B**) RAAS-inhibitors. Percentages refer to changes in the rates of pharmacological treatments from 2005/2006 to 2016/2017. *p* int. refers to the interaction between year of admission and risk group on the utilization of pharmacological treatments. Odds ratios (OR; with 95% confidence intervals) describe the adjusted associations of the year of admission (2005/2006 vs. 2016/2017) with pharmacological treatments. Only in-hospital survivors had been considered. *RAAS* renin–angiotensin–aldosterone-system.

**Figure 5:**
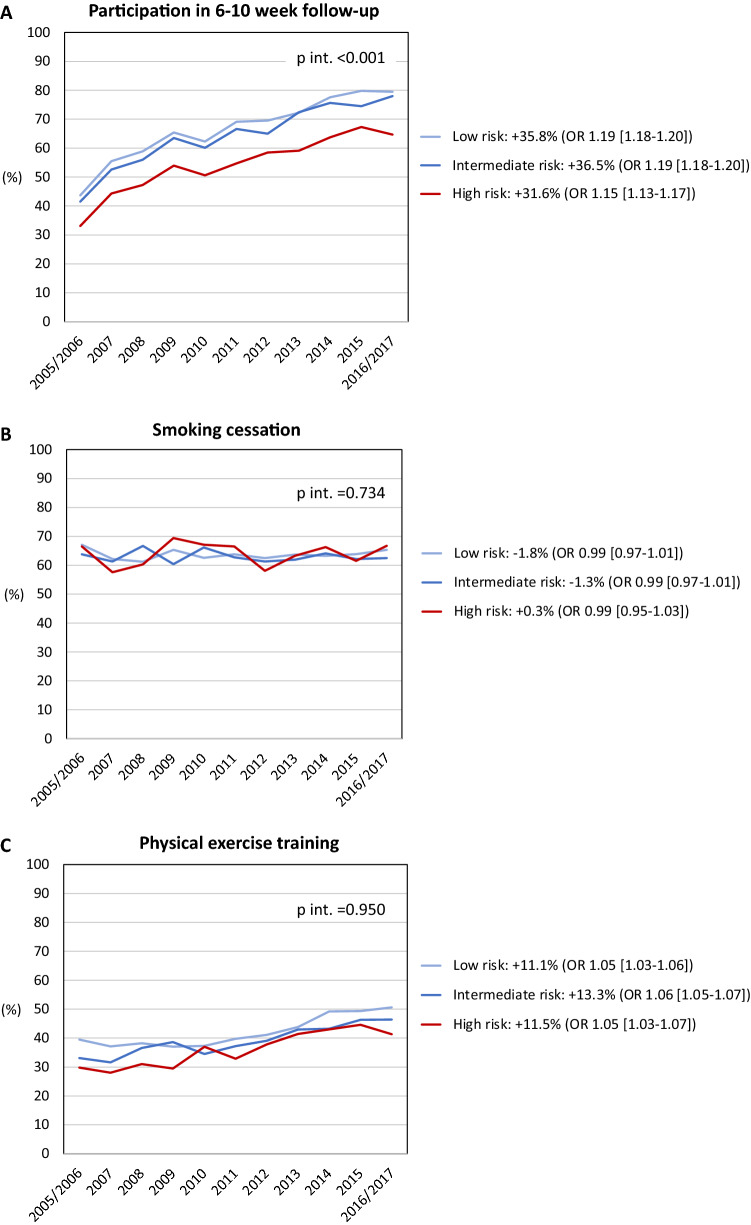
Temporal changes in follow-up and secondary preventive measures. (**A**) participation in the 6–10 week follow-up; (**B**) self-reported smoking cessation; (**C**) participation in exercise training. Percentages refer to changes in the rates of follow-up participation and achievement of secondary preventive measures from 2005/2006 to 2016/2017. *p* int. refers to the interaction between year of admission and risk group on the utilization of coronary interventions. Odds ratios (OR; with 95% confidence intervals) describe the adjusted associations of the year of admission (2005/2006 vs. 2016/2017) with coronary interventions. Only patients eligible for the 6–10 week follow-up had been considered.

The temporal trends for medical interventions are depicted in Figs. 3, 4 and 5. Overall intervention rates increased when comparing the two first with the two last years of the observation period. The only exception was smoking cessation with an overall low rate of 36.5%. A trend towards a more pronounced temporal increase of P2Y12-medication was noted in high-risk patients compared to lower-risk patients (+ 22.2% vs. + 7.8%), as demonstrated by the interaction analysis (*p*_interaction_ < 0.001) and non-overlapping 95% CI of the OR comparing the two first with the two last years of the observation period. In contrast, temporal increases were less pronounced in high-risk patients for RAAS-inhibitor medication (+ 8.5% vs. + 13.0%) and participation in the 6–10 week follow-up (+ 31.6% vs. + 36.3%); *p*_interaction_ < 0.001 for both. The temporal changes in the other investigated interventions did not differ among risk cohorts.

Restricting these analyses to patients aged < 80 years (n = 127,748 following exclusions) revealed similar, albeit partly weaker associations between high risk, medical interventions and their temporal trends (Supplementary Tables S3 and S4; Supplementary Figs. S1 and S2). However, a significant temporal trend towards more frequent in-hospital PCI/CABG was noted in high-risk patients compared to lower-risk patients (+ 23.6% vs. + 12.5%); *p*_interaction_ < 0.001. Discharge medication with RAAS-inhibitors was not considered in this subanalysis since the prescription rate was 87.0% (Supplementary Table S4) and exceeded the predefined utilization benchmark.

## Discussion

Several important observations were made in this investigation. First, the GRACE 2.0 score provided excellent prognostic accuracy regarding 1-year all-cause mortality. These results can be regarded as an external validation and are assuring given the emphasis on the score in current European guidelines^8^. Second, the rates of almost all medical interventions increased progressively over time including patients at high risk according to the GRACE 2.0 score. Third, despite these improvements, utilization rates were lower in high-risk patients compared to those at lower-risk. The only exception was treatment with betablockers with a medication rate of 86.4% in high-risk patients.

### Coronary procedures

Compared to lower-risk patients, those at high risk were less likely to receive early reperfusion in case of STEMI, coronary angiography ≤ 3 days in case of NSTEMI and in-hospital PCI/CABG overall. Lower revascularization rates have been described previously in high-risk cohorts with MI^4–6^. These and our findings may be explained by a disinclination to investigate these patients because of perceived risks of peri-interventional complications^11^. Data from randomized controlled trials and registries however, suggest that high-risk patients have more to gain from coronary revascularization^5,6,12^. Accordingly, invasive assessment within 24 h is recommended in high-risk patients defined by a previous iteration of the GRACE score^8,13^. The underlying scientific evidence however, is limited, and this recommendation is not uncontroversial^14^.

In this context, it should be noted that 56.9% of high-risk patients from our cohort underwent coronary angiography. This is a higher rate compared to data presented elsewhere^4,5^. Moreover, the proportion of high-risk patients treated with PCI/CABG increased by + 24.2% during the observation period. In high-risk patients aged < 80 years, interaction analysis indicated a stronger temporal trend regarding more frequent coronary interventions compared to lower-risk patients aged < 80 years. This is encouraging but emphasizes also that any decision towards coronary procedures needs to be individualized in elderly high-risk patients from the perspective of a balanced risk/benefit ratio. As to whether these findings still can be regarded indicative of a treatment paradox could be discussed. Nonetheless, the temporal trends indicate that the clinical dimension of these disparities appears to be diminishing.

### Pharmacological treatments

Medication rates of betablockers and statins were generally high and exceeded the benchmarks specified in the SWEDEHEART quality index. RAAS-inhibitors in contrast, tended to be less often prescribed in high-risk patients and medication rates increased more slowly over time as compared to lower-risk patients. Again, this was mainly driven by a lower use of RAAS-inhibitors in the elderly. Excluding patients aged ≥ 80 years raised the overall medication rate to > 85%. Notably, ACE-inhibition has been shown to lower mortality in MI patients at advanced age^15,16^, an entity contributing to higher GRACE 2.0 points. While caution is required in treating older patients due to greater risk of adverse side-effects, we want to emphasize that those with obvious treatment contraindications (i.e. eGFR < 20 mL/min/1.73 m^2^) or without a clear indication (i.e. left-ventricular ejection fraction > 0.50 without concomitant diabetes, hypertension or known heart failure) had not been considered in our analysis. Accordingly, our data indicate the persisting presence of an undertreatment with RAAS-inhibitors, in particular in the elderly, and intriguingly without a reduction of this disparity over time.

For P2Y12-blockers, medication rates started at high levels at the beginning of the observation period and progressed constantly in the three risk groups. Encouragingly, this increase was most pronounced in high-risk patients, possibly related to more frequent revascularization procedures in these patients.

### Follow-up and secondary preventive measures

The overall participation rate in the 6–10 week follow-up documented in SEPHIA increased constantly during the observation period. However, this was less pronounced in the high-risk group. Some of these patients may have decided not to attend follow-up because of comorbidities, frailty or other inconveniences such as long travelling distances in rural Sweden. Other patients may have been perceived not suitable for structured follow-up, in particular during the early years when SEPHIA had not been fully implemented at all hospitals participating in SWEDEHEART. However, among patients who actually attended follow-up, those at high risk still were less likely to participate in exercise training and to stop smoking. We acknowledge that our findings inevitably are affected by selection and survival bias. Given the salutary benefits from secondary preventive strategies^17^, our data nonetheless, emphasize the importance of further efforts to improve follow-up participation rates in high-risk patients, e.g. by health education initiatives or remote monitoring using telephone- or web-based solutions.

### Limitations

Our study has limitations that need to be considered. Although all hospitals participating in SWEDEHEART are annually monitored, the data cannot be of the same quality as in a prospective observational study. However, the accuracy of the data and the registry has been reported to be high^18^. While the SWEDEHEART framework recommends the use of criteria outlined in the Universal Definition^19^, there was no independent adjudication of the discharge diagnosis of MI. This implies some risk of misclassification. SWEDEHEART is restricted to patients admitted to CCUs. Extrapolating our findings to MI patients managed in other facilities should thus, be done with caution. We did not consider patients discharged with a diagnosis of unstable angina since quality/performance measures incorporated in the SWEDEHEART index only take MI patients into consideration. This also applies to structured follow-up documentation in the SEPHIA registry. We did not account for the effects of methodological improvements during the observation period, e.g. the switch from femoral to radial puncture site, use of newer generation drug-eluting stents, different regimes of antiplatelet therapy or high-dose statin treatment. The GRACE 2.0 score is based on several prognostic indicators, some of which reflecting acute risk, others chronic risk. Since the score is an integrative estimate of these entities, we are unable to disentangle their individual contribution to the utilization of medical interventions. Data on early reperfusion was missing in 12.6% of treated STEMI patients, representing a cohort with several high-risk features. Accordingly, treatment disparities in this regard may have been underestimated. Finally, there may have been unmeasured confounders not documented in SWEDEHEART (e.g. patient refusal, comorbidities, frailty or short expected survival) that could have influenced management decisions. This applies in particular to coronary procedures.

## Conclusions

Management in MI has improved during the past decades^1–3^. Even for high-risk patients, rates of medical interventions are progressing over time which is encouraging. However, risk-associated management disparities still exist with smaller temporal increases in high-risk patients for RAAS-inhibitor medication and participation in structured follow-up, interventions with well-acknowledged prognostic benefit. While the latter may be affected by issues not captured in the SWEDEHEART registry, the underutilization of RAAS-inhibitors in the elderly concern, in particular in the light of increasing longevity in Sweden. Whenever possible, the risks and benefits of this and other less utilized treatments should thus, be carefully weighed before withholding them in patients identified as having high risk. Our findings also emphasize the need of further quality improvement initiatives to reduce the dimension of evidence-to-practice gaps.

## Supplementary Information

Supplementary Information.

## Data Availability

The dataset analyzed in this study is not publicly available due to Swedish patient privacy and secrecy laws regulating access to SWEDEHEART, and due to ethical restrictions. However, access can be made available at Uppsala Clinical Research Center upon reasonable request and under the provision that the data is accessed onsite and does not leave Uppsala University.
