# Supplementary material for: Risk-associated management disparities in acute myocardial infarction
Source: Sci Rep. 2021 Dec 29;11:24488. doi: 10.1038/s41598-021-03742-6 (PMC8716523; doi:10.1038/s41598-021-03742-6)
Supplement: Supplementary file 1 — Supplementary Information. [file 41598_2021_3742_MOESM1_ESM.docx]

**SUPPLEMENTARY DATA**

**Supplementary Table S1. Clinical characteristics in relation to GRACE 2.0 score categories in patients participating in the 6-10 week follow-up.**

**Supplementary Table S2A. Clinical characteristics and outcome of STEMI patients with and without available data on reperfusion time.**

**Supplementary Table S2B. Clinical characteristics and outcome of NSTEMI patients with and without available data on time interval to invasive coronary angiography.**

**Supplementary Table S3. Medical interventions in relation to GRACE 2.0 score categories in patients aged <80 years.**

**Supplementary Table S4. Utilization of medical interventions in high-risk patients aged <80 years.**

**Supplementary Figure S1. Temporal changes in coronary interventions in patients aged <80 years. A) early reperfusion in STEMI; B) coronary angiography ≤3 days in NSTEMI; C) in-hospital PCI/CABG.**

**Supplementary Figure S2. Temporal changes in P2Y12-blocker treatment at discharge in patients aged <80 years.**

**Supplementary Table S1. Clinical characteristics in relation to GRACE 2.0 score categories in patients participating in the 6-10 week follow-up.**

|  | **Low risk (n=26,315)** | **Intermediate risk (n=27,778)** | **High risk (n=6993)** | **Missing data** | **Total cohort (n=61,086)** |
| --- | --- | --- | --- | --- | --- |
|  |  |  |  |  |  |
|  |  |  |  |  |  |
| **Risk factors*** |  |  |  |  |  |
| Age (years) | 57 (51-63) | 67 (62-71) | 70 (65-72) | - | 64 (57-69) |
| Men | 20,198 (76.8%) | 20,117 (72.4%) | 5085 (72.7%) | - | 45,400 (74.3%) |
| Current smoking | 8944 (34.0%) | 7503 (27.0%) | 1849 (10.1%) | 1 | 18,296 (30.0%) |
| Hypertension | 9741 (37.0%) | 12,537 (48.3%) | 3691 (52.8%) | 1 | 25,969 (42.5%) |
| Diabetes | 3386 (12.9%) | 5127 (18.5%) | 1879 (26.9%) | 2 | 10,392 (17.0%) |
| Hyperlipidemia | 5080 (19.3%) | 7152 (25.8%) | 2350 (33.6%) | 36 | 14,582 (23.9%) |
| Body mass index (kg/m^2^) | 27.5 (25.1-30.5) | 26.9 (24.5-29.7) | 26.8 (24.3-30.0) | 5273 | 27.2 (24.7-30.1) |
| eGFR (mL/min/1.73m^2^) | 93.0 (83.1-100.4) | 83.1 (69.9-92.3) | 63.1 (48.0-81.5) | - | 86.9 (72.7-95.9) |
|  |  |  |  |  |  |
| **Comorbidities*** |  |  |  |  |  |
| Heart failure | 315 (1.2%) | 761 (2.7%) | 516 (7.4%) | 1 | 1592 (2.6%) |
| Previous stroke | 598 (2.3%) | 1276 (4.6%) | 543 (7.8%) | 133 | 2417 (4.0%) |
| Peripheral artery disease | 345 (1.3%) | 898 (3.2%) | 522 (7.5%) | - | 1765 (2.9%) |
| COPD | 623 (2.4%) | 1571 (5.7%) | 610 (8.7%) | - | 2804 (4.6%) |
| Previous/present cancer | 188 (0.7%) | 490 (1.8%) | 203 (2.9%) | - | 881 (1.4%) |
|  |  |  |  |  |  |
| **Diagnosis** |  |  |  |  |  |
| NSTEMI | 18,056 (68.6%) | 14,010 (50.4%) | 3115 (44.5%) | - | 35,181 (57.6%) |
| STEMI | 8259 (31.4%) | 13,768 (49.6%) | 3878 (55.5%) | - | 25,905 (42.4%) |
|  |  |  |  |  |  |

* Information based on data obtained upon admission.

eGFR: estimated glomerular filtration rate; COPD: chronic obstructive pulmonary disease; NSTEMI: non-ST-elevation myocardial infarction; STEMI: ST-elevation myocardial infarction.

**Supplementary Table S2A. Clinical characteristics and outcome of STEMI patients with and without available data on reperfusion time.**

|  | **Data on reperfusion time available** | |  |  |
| --- | --- | --- | --- | --- |
|  |  |  |  |  |
|  |  |  |  |  |
|  | **Yes (n=49,743)** | **No (n=15,107)** | **p-value** | **Missing data** |
|  |  |  |  |  |
|  |  |  |  |  |
| **Risk factors** |  |  |  |  |
| Age (years) | 68 (59-77) | 77 (65-85) | <0.001 | - |
| Men | 34,903 (70.2%) | 8758 (58.0%) | <0.001 | - |
| Current smoking | 14,285 (28.7%) | 2889 (19.1%) | <0.001 | 2 |
| Hypertension | 21,206 (42.6%) | 7097 (47.0%) | <0.001 | 1 |
| Diabetes | 7620 (15.3%) | 3007 (19.9%) | <0.001 | 1 |
| Hyperlipidemia | 8905 (17.9%) | 3287 (21.8%) | <0.001 | 13 |
| Body mass index (kg/m^2^) | 26.3 (24.1-29.2) | 25.5 (23.1-28.4) | <0.001 | 10,582 |
| eGFR (mL/min/1.73m^2^) | 81.3 (63.9-93.4 | 67.9 (47.5-85.5) | <0.001 | - |
|  |  |  |  |  |
| **Comorbidities** |  |  |  |  |
| Previous MI | 5291 (10.6%) | 2904 (19.2%) | <0.001 | 1 |
| Previous PCI/CABG | 4157 (8.4%) | 1592 (10.5%) | <0.001 | 1 |
| Heart failure | 1267 (2.5%) | 1250 (8.3%) | <0.001 | 1 |
| Atrial fibrillation at admission | 2994 (6.0%) | 1904 (12.6%) | <0.001 | - |
| Previous stroke | 2628 (5.3%) | 1735 (11.6%) | <0.001 | 716 |
| Peripheral artery disease | 1482 (3.0%) | 905 (6.0%) | <0.001 | - |
| COPD | 2493 (5.0%) | 1154 (7.6%) | <0.001 | - |
| Previous/present cancer | 938 (1.9%) | 561 (3.7%) | <0.001 | - |
|  |  |  |  |  |
| **1-year mortality** | 4797 (9.6%) | 4041 (26.7%) | <0.001 | - |
|  |  |  |  |  |

STEMI: ST-elevation myocardial infarction; eGFR: estimated glomerular filtration rate; MI: myocardial infarction; PCI: percutaneous coronary intervention; CABG: coronary artery bypass grafting; COPD: chronic obstructive pulmonary disease.

**Supplementary Table S2B. Clinical characteristics and outcome of NSTEMI patients with and without available data on time interval to invasive coronary angiography.**

|  | **Data on time interval to coronary angiography available** | |  |  |
| --- | --- | --- | --- | --- |
|  |  |  |  |  |
|  |  |  |  |  |
|  | **Yes (n=70,611)** | **No (n=28,531)** | **p-value** | **Missing data** |
|  |  |  |  |  |
|  |  |  |  |  |
| **Risk factors** |  |  |  |  |
| Age (years) | 69 (61-76) | 84 (77-88) | <0.001 | - |
| Men | 48,156 (68.2%) | 14,579 (51.1%) | <0.001 | - |
| Current smoking | 14,428 (20.4%) | 2780 (9.7%) | <0.001 | 2 |
| Hypertension | 35,844 (50.8%) | 15,891 (55.7%) | <0.001 | 1 |
| Diabetes | 14,931 (21.1%) | 7580 (26.6%) | <0.001 | 2 |
| Hyperlipidemia | 22,103 (31.3%) | 8766 (30.7%) | 0.073 | 40 |
| Body mass index (kg/m^2^) | 26.9 (24.4-29.9) | 25.0 (22.5-28.1) | <0.001 | 15,474 |
| eGFR (mL/min/1.73m^2^) | 79.8 (63.6-91.6 | 56.0 (40.5-74.3) | <0.001 | - |
|  |  |  |  |  |
| **Comorbidities** |  |  |  |  |
| Previous MI | 12,926 (18.3%) | 9152 (32.1%) | <0.001 | - |
| Previous PCI/CABG | 11,983 (17.0%) | 5165 (18.1%) | <0.001 | - |
| Heart failure | 3676 (5.2%) | 5331 (18.7%) | <0.001 | - |
| Atrial fibrillation at admission | 5659 (8.0%) | 6638 (23.3%) | <0.001 | 2 |
| Previous stroke | 4663 (6.6%) | 5033 (17.7%) | <0.001 | 149 |
| Peripheral artery disease | 3247 (4.6%) | 2985 (10.5%) | <0.001 | - |
| COPD | 4651 (6.6%) | 3520 (12.3%) | <0.001 | - |
| Previous/present cancer | 1668 (2.4%) | 1636 (5.7%) | <0.001 | - |
|  |  |  |  |  |
| **1-year mortality** | 3598 (5.1%) | 9569 (33.5%) | <0.001 | - |
|  |  |  |  |  |

NSTEMI: non-ST-elevation myocardial infarction; eGFR: estimated glomerular filtration rate; MI: myocardial infarction; PCI: percutaneous coronary intervention; CABG: coronary artery bypass grafting; COPD: chronic obstructive pulmonary disease.

**Supplementary Table S3. Medical interventions in relation to GRACE 2.0 score categories in patients aged <80 years.**

| **Risk category** | **Low risk** | **Intermediate risk** | **High risk** | **Total cohort** | **Missing data** | **Ex-clusions** |
| --- | --- | --- | --- | --- | --- | --- |
|  |  |  |  |  |  |  |
|  |  |  |  |  |  |  |
| **Coronary procedures** |  |  |  |  |  |  |
| Early reperfusion (STEMI) | 7058 (65.8%) | 13,347 (66.7%) | 6325 (62.9%) | 26,730 (65.6%) | 8845 | - |
| ICA ≤3 days (NSTEMI) | 19,367 (77.8%) | 18,177 (71.4%) | 5716 (62.1%) | 43,260 (72.6%) | 9087 | 6183 |
| In-hospital PCI/CABG | 28,249 (75.1%) | 36,187 (71.5%) | 14,495 (57.8%) | 78,931 (69.6%) | - | 14,376 |
|  |  |  |  |  |  |  |
| **Pharmacological treatments*** |  |  |  |  |  |  |
| P2Y12-blockers | 33,564 (89.4%) | 42,892 (85.4%) | 18,383 (75.4%) | 94,839 (84.6%) | 96 | 12,263 |
| Betablockers | 35,921 (90.0%) | 48,816 (90.7%) | 23,958 (89.8%) | 108,695 (90.3%) | 96 | 3983 |
| RAAS-inhibitors | 16,646 (88.1%) | 28,961 (88.0%) | 15,741 (84.1%) | 61,348 (87.0%) | 96 | 53,857 |
| Statins | 39,411 (95.6%) | 51,828 (93.5%) | 23,934 (86.3%) | 115,173 (92.6%) | 96 | - |
|  |  |  |  |  |  |  |

* Assessed in in-hospital survivors (n=124,485).

STEMI: ST-elevation myocardial infarction; NSTEMI: non-ST-elevation myocardial infarction; PCI: percutaneous coronary intervention; CABG: coronary artery bypass grafting; RAAS: renin-angiotensin-aldosterone-system.

**Supplementary Table S4. Utilization of medical interventions in high-risk patients aged <80 years.**

|  | **n** | **OR (95% CI)** | **p** |
| --- | --- | --- | --- |
|  |  |  |  |
|  |  |  |  |
| **Coronary procedures** |  |  |  |
| Early reperfusion (STEMI) | 40,334 | 0.94 (0.89-0.99) | 0.013 |
| ICA ≤3 days (NSTEMI) | 59,478 | 0.68 (0.64-0.71) | <0.001 |
| In-hospital PCI/CABG | 97,286 | 0.79 (0.76-0.82) | <0.001 |
|  |  |  |  |
| **Pharmacological treatments*** | | |  |
| P2Y12-blockers | 111,941 | 0.69 (0.66-0.71) | <0.001 |
|  |  |  |  |

Odds ratios refer to comparisons of high-risk patients with low- and intermediate-risk patients, considered as one group.

Analysis adjusted for hospital, admission year, sex, current smoking, diabetes, congestive heart failure, previous myocardial infarction, previous percutaneous coronary intervention/coronary artery bypass grafting, previous stroke, atrial fibrillation upon admission, chronic obstructive pulmonary disease, previous or present cancer, peripheral vascular disease, coronary findings (in-hospital PCI/CABG only) and in-hospital PCI/CABG (P2Y12-blockers only).

* Assessed in in-hospital survivors (n=124,485).

OR: odds ratio; CI: confidence interval; STEMI: ST-elevation myocardial infarction; ICA: invasive coronary angiography; NSTEMI: non-ST-elevation myocardial infarction; PCI: percutaneous coronary intervention; CABG: coronary artery bypass grafting.

**Supplementary Figure S1. Temporal changes in coronary interventions in patients aged <80 years. A) early reperfusion in STEMI; B) coronary angiography ≤3 days in NSTEMI; C) in-hospital PCI/CABG.**

| **A)** | **** |
| --- | --- |
| **B)** | **** |
| **C)** | **** |

Percentages refer to changes in the rates of coronary interventions from 2005/2006 to 2016/2017.

P int. refers to the interaction between year of admission and risk group on the utilization of coronary interventions. Odds ratios (OR; with 95% confidence intervals) describe the adjusted associations of the year of admission (2005/2006 vs. 2016/2017) with coronary interventions.

STEMI: ST-elevation myocardial infarction; NSTEMI: NSTEMI: non-ST-elevation myocardial infarction; PCI: percutaneous coronary intervention; CABG: coronary artery bypass grafting.

**Supplementary Figure S2. Temporal changes in P2Y12-blocker treatment at discharge in patients aged <80 years.**

| **** |
| --- |

Percentages refer to changes in the rates of pharmacological treatments from 2005/2006 to 2016/2017.

P int. refers to the interaction between year of admission and risk group on the utilization of pharmacological treatments. Odds ratios (OR; with 95% confidence intervals) describe the adjusted associations of the year of admission (2005/2006 vs. 2016/2017) with pharmacological treatments. Only in-hospital survivors had been considered.
